# Recent Progress in Heavy Metal Ion Decontamination Based on Metal–Organic Frameworks

**DOI:** 10.3390/nano10081481

**Published:** 2020-07-29

**Authors:** Yajie Chen, Xue Bai, Zhengfang Ye

**Affiliations:** 1 Key Laboratory of Integrated Regulation and Resource Development on Shallow Lake, Ministry of Education, College of Environment, Hohai University, Nanjing 210098, China; cyj@hhu.edu.cn; 2 Department of Environmental Engineering, Peking University, Key Laboratory of Water and Sediment Sciences, Ministry of Education, Beijing 100871, China; yezhengfang101411@pku.edu.cn

**Keywords:** heavy metal ions, metal–organic frameworks, adsorption

## Abstract

Heavy metals are inorganic pollutants which pose a serious threat to human and environmental safety, and their effective removal is becoming an increasingly urgent issue. Metal–organic frameworks (MOFs) are a novel group of crystalline porous materials, which have proven to be promising adsorbents because of their extremely high surface areas, optimizable pore volumes and pore size distributions. This study is a systematic review of the recent research on the removal of several major heavy metal ions by MOFs. Based on the different structures of MOFs, varying adsorption capacity can be achieved, ranging from tens to thousands of milligrams per gram. Many MOFs have shown a high selectivity for their target metal ions. The corresponding mechanisms involved in capturing metal ions are outlined and finally, the challenges and prospects for their practical application are discussed.

## 1. Introduction

With the rapid development of industrialization, environmental pollution is becoming an increasingly serious problem, especially heavy metal pollution, which is a widespread cause of concern. The main sources of heavy metals include the electroplating, mining, smelting, battery manufacturing, textile printing and the leather industries [1,2]. The common heavy metal contaminants include arsenic (As), lead (Pb), mercury (Hg), cadmium (Cd) and chromium (Cr). These heavy metals are difficult to degrade and easily accumulate in organisms within the ecosystem, which can directly or indirectly present a risk to humans through drinking water, skin contact and the food chain. Therefore, establishing effective methods to effectively remove toxic heavy metal ions from the environment has become a crucial research aim.

In recent decades, various methods have been adopted to remove heavy metals from wastewater, including chemical precipitation, ion exchange, adsorption, photocatalysis, membrane filtration, electrodialysis, coagulation and flocculation, flotation and electrochemical treatment [3,4]. Among these, adsorption is one of the most commonly applied methods due to its convenience, low-cost and high efficiency for the removal of heavy metal ions [5]. Common adsorbents, such as zeolites, activated carbon, carbon nanotubes, and metal oxides have been widely applied in the adsorption of heavy metal ions. Nevertheless, these adsorbent materials generally suffer from low adsorption capacities, poor adsorption selectivity and unsatisfactory regeneration capabilities [6]. Consequently, the development of novel improved adsorbents is greatly needed, to overcome the existing limitations and maximize their adsorption capacity.

**Figure 1:**
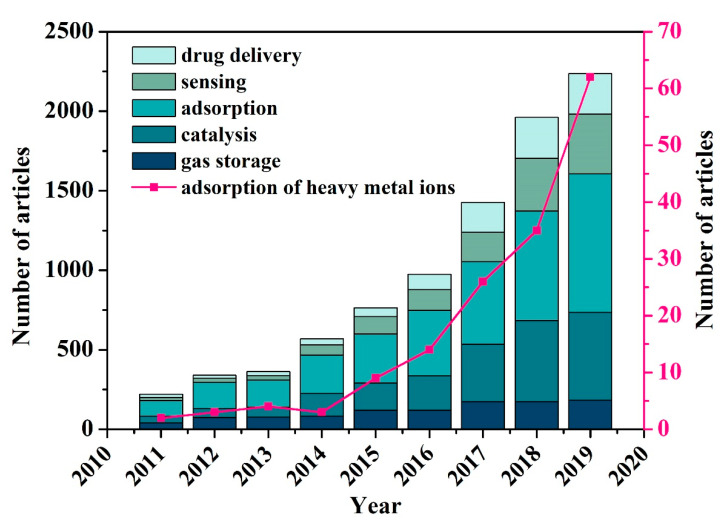
Number of articles retrieved in terms of the subjects “application of metal–organic frameworks in gas storage/catalysis/adsorption/sensing/drug delivery” and “adsorption of heavy metal ions by metal–organic frameworks”, from 2011 to 2019 on Web of Science.

In recent years, metal–organic framework (MOF) materials have attracted much attention due to their wide range of potential applications [7]. They are usually self-assembled through strong coordination bonds between inorganic metal ions/clusters and organic ligands, forming periodic network structures. The characteristics of MOFs such as large surface areas, permanent porosity, multi-functionalization, changeable structures and open metal sites, are beneficial to a wide range of applications, including gas storage [8,9,10,11], separation [12,13,14,15,16], catalysis [17,18,19,20,21], sensing [22,23], drug delivery [24,25,26], bioimaging [27,28], and proton conduction [29,30]. Recently, MOFs have been shown to be effective in the adsorption of toxic pollutants, such as hazardous metal ions and organic compounds [31,32,33,34]. The number of articles related to the application of MOFs in different fields from 2011 to 2019 is shown in Figure 1, according to the statistics provided by the Web of Science, showing an increasing trend in the research on the use of MOFs for the adsorption of heavy metal ions. Many excellent review articles have been published on the removal of heavy metal pollutants from water by MOFs [35,36,37,38]. However, few reviews are written for heavy metal ions specially. Wang et al. [39] systematically studied MOFs’ characteristic structures, application performance and interaction mechanisms in aquatic arsenic removal. Mon et al. [40] mainly introduced the recent development of MOFs as adsorbents for various inorganic and organic pollutants. The most recent review by Rasheed et al. [41] focused on the advance in applications of MOFs to remove organic and inorganic pollutants through adsorption and photocatalysis from wastewater. However, the content of the removal of heavy metal ions by MOFs is not comprehensive. Some of the influencing factors, interaction mechanisms and reusability in the adsorption process were not discussed in detail or the available data were not presented in a clear and accessible manner.

Therefore, this review highlights the recent advances in the application of water-stable MOFs and MOF-based materials for the removal of heavy metal ions, with many representative examples and a detailed discussion of the influencing factors and mechanisms of interaction [42]. The challenges and future prospects for the application of MOFs in this field are also discussed. We hope this review will provide a broad view of MOFs’ application in capturing various types of heavy metal ion pollutants and promote further investigations into the practical application of MOF-based materials for environmental pollution management.

## 2. Recent Studies on MOFs for the Removal of Heavy Metal Ions

### 2.1. Adsorptive Removal of Arsenic

Arsenic is a highly hazardous contaminant which is toxic to living organisms. In surface waters, arsenic exists in the form of As(III) and As(V). The toxicity of inorganic arsenic is higher than that of organic arsenic and generally, arsenic exists in the dissolved inorganic forms of arsenic acids (H_3_AsO_4_), arsenic acids (H_3_AsO_4_) and their related ionic compounds, depending on the acidity/basicity of water. [43]. While both these forms are toxic, As(III) is about 60-fold more poisonous than As(V).

#### 2.1.1. ZIF Family MOFs for Arsenic Adsorption

**Figure 2:**
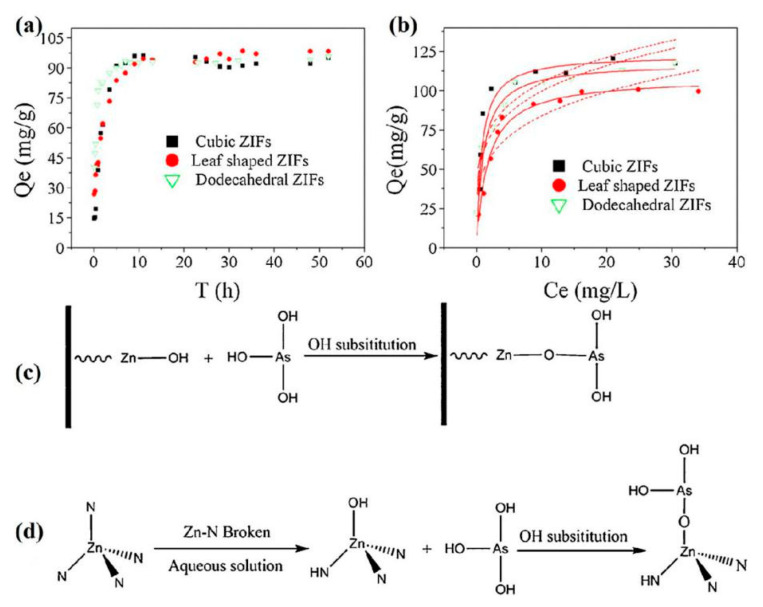
As(III) adsorption (**a**) kinetics (initial arsenic concentration of 35.4 mg L^−1^) and (**b**) isotherms (initial arsenic concentration of 5–70 mg L^−1^) for the three zeolitic imidazolate framework (ZIF) structures. The solid line represents the Langmuir fitting curve, dotted line represents the Freundlich fitting curve; (**c**,**d**) show the proposed pathway for As(III) adsorption on ZIFs. Reproduced with permission from [46]. Copyright Elsevier, 2015.

In recent years, MOFs and MOF-based composites have increasingly been applied in the removal of As(III) and As(V) from water, due to their excellent adsorption capacity. Zeolitic imidazolate frameworks (ZIFs) are a member of the crystalline porous framework family and ZIF-8 in particular has been used to adsorb As(V) [44,45]. The adsorption capacity of ZIF-8 for trace As(V) at a low equilibrium concentration of 9.8 µg L^−1^ was 76.5 mg g^−1^ [44]. Based on a surfactant–amino acid co-templating strategy, Wu et al. [45] prepared hierarchical ZIF-8 with dual micro-/meso-porosity in an aqueous solution at room temperature. The hierarchically structured ZIF-8 exhibited a higher adsorption capacity (90.92 mg g^−1^) than non-modified ZIF-8 synthesized in H_2_O and methanol. The increased adsorption capacity may be due to two possible reasons: (i) the improved utility of the external surface of ZIF-8 crystals with mesostructured pores, and (ii) enhanced porosity, which facilitates mass transfer within the pore structure and increases the contact area for As(V) adsorption. Three different morphological structures of ZIFs-8 (cubic, leaf-shaped and dodecahedral) were compared for the removal of As(III) [46]. The adsorption isotherms of the three adsorbents were generally well fitted by the Langmuir model. Almost 95% of As(III) was removed from the aqueous solution by all three ZIF-8 structures within 10 h (Figure 2a), with maximum adsorption capacities of 122.6, 108.1, and 117.5 mg g^−1^ for the cubic, leaf-shaped, and dodecahedral ZIF-8, respectively (Figure 2b). It is of note, that the As(III) adsorption capacity and kinetics of non-porous leaf-shaped ZIF-8 were similar to those of highly porous cubic and dodecahedral ZIF-8. Based on the results of FTIR and XPS analysis, the adsorption mechanism was attributed to Zn–OH groups, generated from unsaturated zinc atoms and broken Zn–N bonds, adsorbing As(III) through –OH substitution, resulting in the formation of Zn–O–As (Figure 2c,d).

Combining MOFs with other materials to form composites is also a method for As(III) removal. Jian et al. [47] successfully synthesized novel one-dimensional (1D) *β*-MnO_2_@ZIF-8 nanowires (NWs) using a rapid method, achieving simultaneous oxidation and the adsorptive removal of As(III). The kinetic and isotherm data for As(III) adsorption were well fitted using the pseudo-second-order and Langmuir models, respectively. Results showed that MnO_2_@ZIF-8 exhibited a high As(III) adsorption capacity of 140.27 mg g^−1^, which was about 1.6-fold higher than that of pristine ZIF-8 nanoparticles (90.8 mg g^−1^). The increase in As(III) uptake by the composite material was attributed to the oxidation capability of MnO_2_ NWs in the composite core, resulting in the generation of negatively charged As(V), which is more easily adsorbed onto the surface of positively charged ZIF-8 via electrostatic attraction than uncharged As(III) at a pH of 7. Furthermore, the easy separation of *β*-MnO_2_@ZIF-8 from water could be achieved after settling for 30 min under gravity. However, further investigations are required on the selectivity, reusability and the adsorption efficiency under varying pH conditions of this composite.

Additionally, ZIF-8 has also been shown to be efficient for the simultaneous removal of As(V) and As(III), due to the presence of both hydroxyl and amine groups [48]. The time required to reach the adsorption equilibrium for As(V) was only 7 h while for As(III) it was 13 h. The maximum adsorption capacities for As(III) and As(V) were determined using the Langmuir model to be 49.49 mg g^−1^ and 60.03 mg g^−1^, respectively. Arsenic adsorption was not affected by the presence of co-existing anions (i.e., SO_4_^2−^ and NO_3_^−^), although it was influenced significantly by PO_4_^3−^ and CO_3_^2−^. XPS and FTIR analysis indicated that electrostatic attraction and the formation of arsenic complexes with hydroxyl and amine groups on the adsorbent surface may be involved in the adsorption process.

#### 2.1.2. Materials of Institute Lavoisier (MIL) Family MOFs for Arsenic Adsorption

The MIL family of MOFs have also been applied to As(V) adsorption. MIL-53(Fe) displayed fast As(V) adsorption kinetics at three different initial concentrations, requiring only 60–150 min to reach equilibrium due to the Lewis acid–base and electrostatic interactions between H_2_AsO_4_^−^ and Fe^3+^ cations at a pH of 5 [49]. Compared with the iron-1,3,5-benzenetricarboxylic (Fe-BTC) polymers reported by Zhu et al. [50], and the arsenic adsorption capacity of MIL-53(Fe) was improved, although overall the adsorption capacity remained low. MIL-53(Al) exhibited a relatively high adsorption capacity over a solution pH range of 6–9, reaching a maximum of 105.6 mg g^−1^ at pH 8.0 [51]. The effect of several coexisting anions on the adsorption capacity of MIL-53(Al) were also assessed, including Cl^−^, F^−^, NO_3_^−^, SO_4_^2−^, and PO_4_^3−^. Results indicated that only the presence of PO_4_^3−^ exerted a significant influence on the performance of MIL-53(Al), probably due to the similar nature of PO_4_^3−^ and AsO_4_^3−^ and their competition for binding sites. MIL-100(Fe) was reported to have a high AsO_4_^3−^ adsorption capacity of 110 mg g^−1^, exhibiting good reusability and excellent selectivity in the presence of Cl^−^, SO_4_^2−^, NO_3_^−^ and CO_3_^2−^ [52]. Strong Fe–O–As(V) interactions accounted for the adsorption of arsenate by MIL-100(Fe). Furthermore, the adsorption of AsO_4_^3−^ destroyed the long-range order of uniform mesoporous channels in the framework, while it recovered immediately upon AsO_4_^3−^ desorption. This study provided valuable information that advanced our understanding of the long-range order in MOFs and promoted further investigation into MOFs.

Two kinds of magnetic MOF composites, including Fe_3_O_4_@MIL-101 [53] and CoFe_2_O_4_@MIL-100(Fe) [54], have been recently studied for the simultaneous removal of As(III) and As(V). The As(III) and As(V) adsorption capacities of Fe_3_O_4_@MIL-101 were reported to be 121.5 and 80.0 mg g^−1^ at a pH of 7, respectively [53]. Furthermore, the removal efficiency and the selectivity were not affected by the presence of Ca^2+^, Mg^2+^, phosphate ions and natural organic matter. This hybrid material exhibited excellent stability and high affinity towards both As(III) and As(V). CoFe_2_O_4_@MIL-100(Fe) exhibited a fast adsorption rate and high adsorption capacity for As(III) and As(V), due to its nanoscale size and mesoporous structure [54]. The maximum adsorption capacities were 114.8 mg g^−1^ for As(V) and 143.6 mg g^−1^ for As(III). The electrostatic repulsion and size exclusion effects of the MIL-100(Fe) shell resulted in good resistance to interference. The As(V) and As(III) adsorption on this composite material occurred via the substitution of hydroxyl groups with the formation of Fe–O–As bonds, with hydrogen bonding resulting in the multi-layer adsorption of neutral As(III), while the monolayer adsorption of As(V) was observed.

#### 2.1.3. Zr-Based MOFs for Adsorption of Arsenic

Zr-Based MOFs, especially MOF-808 [55] and UiO-66 [56], have also been found to be effective absorbents for the removal of As(V). Li et al. [55] successfully synthesized MOF-808 using a household microwave oven, with an irradiation time of less than 5 min. The arsenic adsorption capacity of the resulting MOF-808 nanoparticles was 24.83 mg g^−1^. In addition, the adsorbent retained 82.10% of its original removal efficiency after five cycles of reuse. UiO-66 was reported to have an ultrahigh adsorption capacity of 303.34 mg g^−1^ at pH 2, which is significantly higher than all other currently available adsorbents (5–280 mg g^−1^, generally less than 100 mg g^−1^) [56]. The superior adsorption capacity of UiO-66 was attributed to its highly porous crystalline structure, containing zirconium oxide clusters that provide a large contact area and numerous active sites, which were thought to be hydroxyl groups and benzenedicarboxylate (BDC) ligands. The arsenic acid (H_3_AsO_4_) could act as acid to release H ions thus binding to the hydroxyl groups in UiO-66 at a pH of 2 and some BDC ligands were exchanged with H_3_AsO_4_, eventually resulting in the formation of arsenic complexes. One disadvantage of UiO-66 was the long adsorption equilibrium time of 48 h, with no data on the potential for reuse reported.

Table 1 lists the recent reports of the use of MOF-based materials for the adsorption of different arsenic species. The adsorption mechanism was mainly ion exchange and coordination with metal nodes of MOFs. Most of the reported MOFs have been different forms of ZIFs and MILs, some of which show excellent selectivity for arsenic under the coexistence of other inorganic anions. However, few MOF-based materials are reusable. Therefore, in future research and practical applications, adsorption equilibrium time and regeneration should be taken into account, as well as the adsorption capacity.

### 2.2. Adsorptive Removal of Chromium

Chromium ions and their corresponding compounds are released from various industrial processes (such as metal plating, tannery, dyes industries) in the form of oxyanions (CrO_4_^2−^, Cr_2_O_7_^2−^ or HCrO_4_^−^) and cations (Cr^3+^).

Li et al. [57] synthesized a cationic silver–triazolate MOF, {[Ag_8_(tz)_6_](NO_3_)_2_·6H_2_O}_n_ (tz^−^ = 3,5-diphenyl-1,2,4-triazolate), capable of the fast, efficient and reversible adsorption of HCrO_4_^−^ via anion exchange, exhibiting a relatively low adsorption capacity of 37 mg g^−1^. Additionally, this MOF exhibited different selectivity performances for Cr(VI) in the presence of different concentrations of several competing anions, such as NO_3_^–^, SO_4_^2–^, ClO_4_^−^, Cl^−^. Overall, the high production costs and low adsorption capacity limit the practical use of this MOF in Cr(VI) removal. Two cationic MOFs (Fujian Institute of Research (FIR)-53 and FIR-54) consisting of nanotube channels were successfully prepared via the assembly of Zn^2+^ and neutral tris(4-(1H-imidazol-1-yl)phenyl)amine (Tipa) ligands [58]. FIR-54 exhibited a higher adsorption capacity for Cr_2_O_7_^2−^ of 103 mg g^−1^ than that of FIR-53 (74.2 mg g^−1^). Both of these two porous materials efficiently trapped Cr_2_O_7_^2−^ via single-crystal-to-single-crystal (SC–SC) transformation. It is of note that FIR-53 showed excellent reversibility and regeneration capacity without losing its uptake capability. Fe-gallic acid MOF is a classic example of a 1D MOF, exhibiting an extremely high Cr_2_O_7_^2−^ uptake capacity of 1709.2 mg g^−1^, attributed to the presence of multiple reaction sites [59]. Under acidic (pH = 2) and alkaline (pH = 11) conditions, the crystalline structure and pore size of this material remained unchanged, resulting in the remarkable removal efficiency over a wide pH range of 3.0–9.0. The selectivity of Fe-gallic acid MOF was almost unaffected by the presence of coexisting anions and metal ions. FTIR and XPS analysis confirmed that the reaction mechanisms between Fe-gallic acid MOF and Cr(VI) were reduction, metal substitution and co-precipitation.

Innovations in the structure and morphology of an adsorbent are a key method for the enhancement of adsorption sites, the density of reaction sites and ultimately the adsorption capacity. A well defined core-double-shell-structured magnetic polydopamine@ZIF-8 (MP@ZIF-8) consisting of a magnetic core, a polydopamine (PDA) inner shell and a porous ZIF-8 outer shell, was fabricated using an environmentally-friendly approach [60]. MP@ZIF-8 exhibited a Cr(VI) adsorption capacity of 136.56 mg g^−1^, with synergistic reduction and adsorption. The adsorption kinetics of Cr(VI) followed the pseudo-second-order model well, suggesting a chemical reaction process. The removal efficiency was highly dependent on the pH conditions. The MP@ZIF-8 composite exhibited good regeneration performance after five cycles of reuse. Importantly, with the reduction of nitrogen atom groups on ZIF-8 and PDA, Cr(VI) was easily converted into lower toxicity Cr(III) and then immobilized on MP@ZIF-8. MOFs combined with alginic acid (metal organic resin (MOR)-1-HA [61,62] and MOR-2-alginic acid (HA) [63]) were shown to be excellent adsorbents for Cr_2_O_7_^2−^. Experimental results showed that Cr_2_O_7_^2−^ was removed by exchange with Cl^−^ in the composites, with MOR-1-HA exhibiting a maximum adsorption capacity of 280 mg g^−1^, which was the highest of the three reported composites. Furthermore, MOR-HA composites were successfully used as a stationary phase in ion-exchange columns, showing high efficiency for the removal of Cr(VI) under a variety of conditions, which is promising for practical application in wastewater treatment.

Different kinds of MOF-based materials were utilized for chromium adsorption (Table 2), with several MOFs exhibiting outstanding adsorption performance. Most investigations have focused on the adsorption of Cr_2_O_7_^2−^, with adsorption capacities ranging from 74 mg g^−1^ to 1709.2 mg g^−1^. In general, the process of aqueous Cr(VI) adsorption by MOFs involved multiple steps, among which anion exchange and adsorption–reduction were the two main adsorption mechanisms.

### 2.3. Adsorptive Removal of Mercury

The main chemical species of mercury are elemental mercury (Hg^0^), inorganic mercury (Hg^2+^) and methylmercury (MeHg). In particular, divalent mercury (Hg^2+^) is an environmental pollutant widely found in surface water and has a significant impact on human health even at lower doses.

#### 2.3.1. Binding with Sulfur Atoms

It is well known that Hg^2+^ ions possess a high affinity for sulfur atoms. To date, various MOFs with sulfur-containing functional groups have been investigated for Hg^2+^ capture. A robust and water-stable, hetero-bimetallic bioMOF with the formula {Ca^II^Cu^II^_6_[(*S*,*S*)-methox]_3_(OH)_2_(H_2_O)}·16H_2_O was reported by Pardo et al. [64], exhibiting an extremely high Hg^2+^ adsorption capacity of 900 mg g^−1^. The process of HgCl_2_ adsorption proceeded via a two-step process that involved the extremely fast initial adsorption of three molecules of HgCl_2_ per formula unit, followed by a slower adsorption process, reaching a final loading of up to five molecules. Additionally, this bioMOF was also able to absorb highly toxic CH_3_Hg^+^ with an adsorption capacity of 166 mg g^−1^. Both of these pollutants were bound by sulfur atoms from the thioether chains present in the channels of the MOF. Following this, the same research group reported a novel water-stable and eco-friendly MOF modified with thioalkyl groups, able to capture HgCl_2_ from aqueous media in an efficient, selective and rapid manner [65]. Due to the strong affinity of S atoms for mercury, this MOF which has the formula {Cu_4_^II^[(*S*,*S*)-methox]_2_}·5H_2_O, could selectively adsorb Hg^2+^ in the presence of other metal cations, including Na^+^, Mg^2+^, Ca^2+^, Al^3+^, and Fe^3+^. Moreover, the concentration of HgCl_2_ decreased from the initial value of 10 mg L^−1^ to below 0.002 mg L^−1^ which is within the permissible limits for drinking water (0.006 mg L^−1^) after 15 min of adsorption. The authors ascribed the selective and efficient capture process to the materials structural features, enabling strong and specific coordinative interactions contributing to the highly stable tetrahedral conformation of the resulting HgCl_2_S_2_ adduct. An SCN^−^ group, containing both a hard N atom and soft S atom, was introduced into the framework, forming a novel sulfur-functionalized MOF, FJI-H12, which exhibited a relatively high Hg^2+^ adsorption capacity of 439.8 mg g^−1^ [66]. This study achieved the large-scale synthesis of FJI-H12 microcrystalline powder at room temperature. Interestingly, the continuous and fast removal of Hg^2+^ from water has also been performed using a column loaded with FJI-H12 microcrystals. If the efficiency of FJI-H12 could be improved after regeneration, this material would show high potential for practical application.

**Figure 3:**
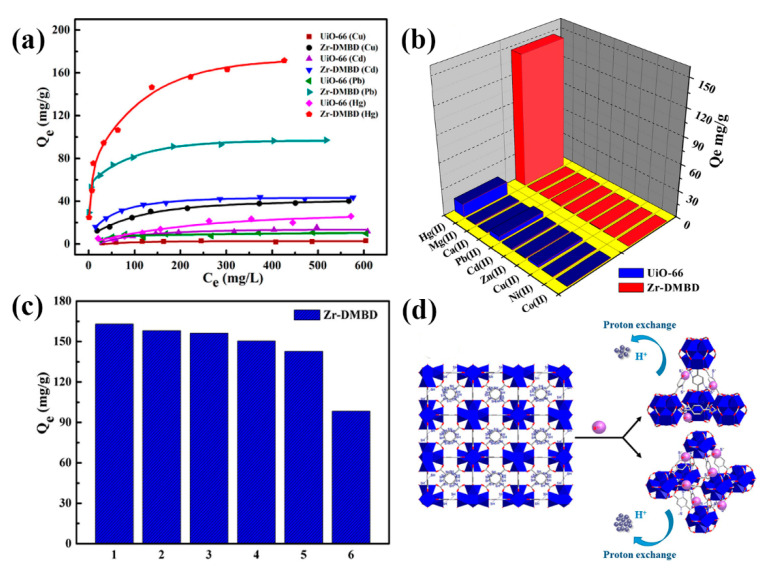
(**a**) Adsorption isotherms for the metal ion adsorption on UiO-66 and Zr-DMBD (prepared using ZrCl_4_ and 2,5-dimercapto-1,4-benzenedicarboxylic acid (H_2_DMBD)); (**b**) the selective adsorption of metal ions by UiO-66 and Zr-DMBD in mixed solutions of nine coexisting metal ions with initial concentrations of 300 mg L^−1^ each; (**c**) the reusability of Zr-DMBD for Hg(II) adsorption; (**d**) Hg(II) coordination with S of thiol groups on Zr-DMBD through a proton exchange reaction mechanism. Reproduced with permission from [67]. Copyright American Chemical Society, 2018.

A thiol-modified Zr-based MOF (Zr-DMBD) with free-standing and accessible thiol groups was prepared using ZrCl_4_ and 2,5-dimercapto-1,4-benzenedicarboxylic acid (H_2_DMBD) [67]. Zr-DMBD exhibited remarkable Hg^2+^ capture capabilities, with a maximum adsorption capacity of 171.5 mg g^−1^, approximately 9-fold higher than that of pristine UiO-66 (Figure 3a), although Zr-DMBD could only adsorb Hg^2+^ when present at a high concentration of 300 mg L^−1^ (Figure 3b). In addition, Zr-DMBD exhibited good regeneration efficiency without any observed decline in adsorption capacity after five cycles of reuse (Figure 3c). Based on a series of characterization assessments and quantitative correlation analysis, the high adsorption capacity and outstanding selectivity of Zr-DMBD was ascribed to the coordination between S^2−^ and Hg^2+^, with thiol group hydrogen atoms being replaced and released into the solution via a proton exchange reaction (Figure 3d). Defective UiO-66 modified with thiol groups (denoted as UiO-66-SH) was achieved using a facile method under mild conditions and utilized for the selective extraction of mercury from aqueous solutions [68]. UiO-66-SH exhibited a fast Hg^2+^ adsorption rate of more than 99% within 20 min and a high pseudo-second-order rate constant of 3.4 × 10^−2^ g/(mg·min), with a maximum adsorption of 3.91 mmol/g. In addition, UiO-66-SH was shown to have a high adsorption performance over a broad pH range (2.3–8.0), retaining a good level of effectiveness (>90%) after seven cycles of regeneration. Moreover, UiO-66-SH displayed selective adsorption towards Hg^2+^ in the presence of coexisting divalent metal ions (i.e., Co^2+^, Cd^2+^, Cu^2+^, Ni^2+^, Ba^2+^ and Mn^2+^), although the Hg^2+^ adsorption rate decreased by an average of 30 and 40% in the presence of Zn^2+^ and Pb^2+^, respectively.

#### 2.3.2. Binding with Other Functional Groups

In addition to combining with sulfur, Hg^2+^ can also combine with other functional groups such as hydroxyl, acylamide and carbonyl groups in the binding sites of MOF channels. Luo et al. [69] prepared a novel MOF material functionalized by both hydroxyl and acylamide groups, achieving an adsorption capacity of approximately 278 mg g^−1^ according to the Langmuir model results. The kinetic data were fitted using the pseudo-second-order kinetic model, implying that the adsorption process was chemisorption. Importantly, this adsorbent could remove 80% of the Hg^2+^ from the solution within 1 h, even with initial Hg^2+^ concentrations as low as 100 ppb. However, this MOF was effective over a limited pH range (4–7) and the selectivity or reusability of this material has not been reported to date.

A novel thymine-functionalized MIL-101 (MIL-101-Thymine) material was synthesized via a post-synthesis method [70]. MIL-101-Thymine was able to selectively remove Hg^2+^ over other metal cations, although a low adsorption capacity of 51.27 mg g^−1^ was reported. The adsorption process was described based on the good fitting of the data using the Langmuir model. XPS results also confirmed that Hg^2+^ coordinated with the N of thymine groups on MIL-101-Thymine. Moreover, MIL-101-Thymine was applied to remove trace Hg^2+^ from real water samples, with satisfactory recoveries achieved. However, 34% of the adsorption capacity of MIL-101-Thymine was lost in the third cycle of reuse. Five amino-based MOFs with high oxidation state central metals (Al^3+^, Zr^4+^, Cr^3+^, Fe^3+^, and Ti^4+^) were successfully fabricated based on the same luminescent organic ligand NH_2_–H_2_BDC and screened for their capacity to simultaneous detect and remove toxic Hg^2+^ [71]. Among these materials, NH_2_-MIL-53(Al) exhibited an excellent capability for Hg^2+^ detection with a wide response interval (1−17.3 μM), low detection limit (0.15 μM), good selectivity, a wide range of pH adaptation (4.0–10.0) and strong resistance to interference. Furthermore, NH_2_–MIL–53(Al) exhibited efficient Hg^2+^ adsorption performance, with fast uptake kinetics (within 60 min) and a large loading capacity of 153.85 mg g^−1^, using strong coordination between the amino groups and Hg^2+^. Additionally, the satisfactory level of water stability and reusability further support the feasibility of use of this material in complex environmental matrices, making NH_2_–ML–53(Al) a promising platform for synchronous Hg^2+^ detection and removal from water.

To improve the features of MOFs and increase their efficiency in the removal of heavy metal ions, Esrafili et al. [72] designed a novel dual-functionalized metal–organic framework (DF-MOF), incorporating different percentages of the ligand *N*1,*N*3-di(pyridine-4-yl) malonamide (S) into urea-containing MOF (TMU-32, TMU = Tarbiat Modares University), forming TMU-32S (with ligand incorporation percentages of 33%, 65% and 100%). Among the three TMU-32S compounds with different percentages of malonamide ligand, TMU-32S–65% demonstrated exceptional Hg^2+^ selectivity. This method of introducing multiple adsorption sites into the MOF backbone resulted in a maximum Hg^2+^ uptake capacity of 1428 mg g^−1^, due to the synergistic effects of both hydrophilic urea and malonamide functional groups.

Table 3 presents the Hg^2+^ adsorption data reported for the MOF-based materials. Due to the strong-binding capability between the sulfur atoms and Hg^2+^, sulfur-containing functional groups are commonly incorporated into the design of MOFs. In order to regulate the adsorption properties, the combination of highly porous structures and well distributed functionalities are the key effective adsorption of Hg^2+^.

### 2.4. Adsorptive Removal of Lead

The release of lead ions from industrial activities such as mining, lead-acid battery production, electroplating and microelectronic production, poses a serious threat to human and environmental health [73,74,75].

#### 2.4.1. Binding with Amino Groups

**Figure 4:**
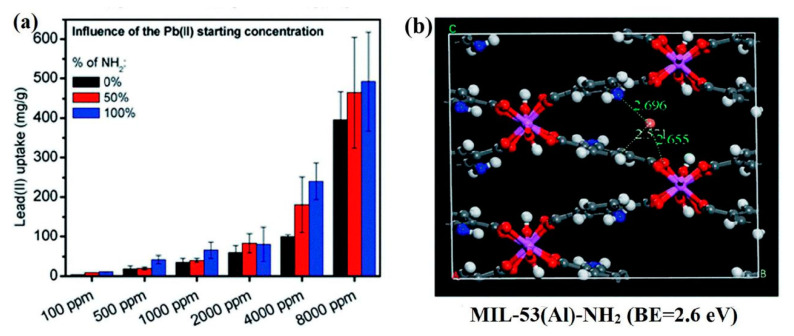
(**a**) Lead(II) uptake in water by magnetic framework composites (MFCs) with different loadings of amino groups, using different initial concentrations of Pb(II) (100 to 8000 ppm); (**b**) the density functional theory (DFT)-optimized locations and binding energies of the lead atom in MIL–53(Al)–NH_2_ calculated using the DFT-D2 method; The distances shown are in angstroms. Color code: C, grey; H, white; N, blue; Al, pink; Pb, orange. Reproduced with permission from [77]. Copyright Royal Society of Chemistry, 2015.

The combination of an ethylenediamine (ED)–NH_2_ group with unsaturated Cr metal centers of MOFs by strong coordination bonds was reported by Luo et al. [76]. The Pb^2+^ adsorption capacity of the modified ED-MIL-101(Cr) was 81.09 mg g^−1^, which is more than 5-fold higher than that of MIL-101. This material exhibited fast adsorption kinetics (30 min) and competitive adsorption studies revealed that ED-MIL-101 had a high selectivity for Pb^2+^. Moreover, ED-MIL-101 was applied to real water samples, achieving a 97.22% removal of Pb^2+^ and was effective over three cycles of reuse with minimal loss in the adsorption capacity. The combination of MOFs and magnetic nanoparticles has shown their high potential as a composite material in practical applications. Amino-functionalized MIL-53 is a typical example of the use of magnetic MOFs for the removal of Pb^2+^, showing a remarkable adsorption capacity of 492.4 mg g^−1^ [77]. Different amounts of amino group loading were found to affect the adsorption capacity (Figure 4a). Furthermore, the magnetism of this composite allowed direct collection from the solution for reuse. The binding energy (2.6 eV) calculated using the density functional theory (DFT) method, confirmed that Pb^2+^ underwent a strong interaction with the amino groups in NH_2_–MIL–53(Al) (Figure 4b).

The novel incorporation of melamine into MOFs was successfully performed, with use as an absorbent for the removal of Pb^2+^ [78]. Compared with the unmodified MOFs (115 mg g^−1^), the adsorption capacity was increased to 205 mg g^−1^ under the conditions of pH 6, at 40 °C, in a 120 min adsorption assay at a low initial Pb^2+^ concentration of 10 mg L^−1^. The adsorption mechanism was confirmed as a coordination interaction between the amino groups (–NH_2_) and Pb^2+^. This research laid the foundation for the successful application of melamine-MOFs in the adsorptive removal of Pb^2+^.

#### 2.4.2. Binding with Other Functional Groups

Ke et al. [79] reported another magnetic core-shell MOF with thiol-containing groups possessing excellent Pb^2+^ selectivity and a calculated adsorption capacity of 215.05 mg g^−1^. This magnetic microsphere can be reused for over four successive cycles, with only a minor loss of Pb^2+^ adsorption efficiency. The MOF-based composite exhibiting the highest Pb^2+^ adsorption capacity (558.66 mg g^−1^) is MCNC@Zn-BTC, which combines magnetic cellulose nanocrystals with a MOF, achieving adsorption equilibrium in only 30 min [80]. Like most reported MOF-based materials, the adsorption of Pb^2+^ fitted well with both the pseudo-second-order kinetic model and Langmuir isotherm model. In addition, the Pb^2+^ removal remained greater than 80% after five cycles of reuse. A novel MOF modified with carbomethoxy groups was constructed for the removal of Pb^2+^ from aqueous solution. The author selected 2,2’-azodibenzoic acid (denoted as H_2_ADB) and (pyridin-3-yl)methyl 4-(2-(4-((pyridin-3-yl)methoxy)-phenyl)diazenyl)benzoate] (denoted as L) as ligands for the assembly of MOF, {[Cd(ADB)L_2_]·1.5DMF·2H_2_O}_n_ [81]. This MOF material exhibited rapid kinetics and a high removal efficiency, even at a low Pb^2+^ concentration of 0.1 ppm. The good adsorption performance was mainly attributed to the significant affinity between the carbomethoxy groups and Pb^2+^, based on FTIR and XPS results. This MOF could selectively adsorb Pb^2+^ in the presence of Na^+^, Mg^2+^, K^+^, Ca^2+^ while Zn^2+^, Co^2+^, Ni^2+^, Cd^2+^ had a significant effect on adsorption. Titanium-based MOF MIL-125 was synthesized via the hydrothermal-solvent method and mixed with chitosan (CS) to form solidified beads for the removal of Pb^2+^ [82]. The synthesized MIL-125-CS beads contained carboxyl and hydroxyl groups derived from chitosan, which played a significant role in the Pb^2+^ adsorption process. It was found that the adsorption of Pb^2+^ onto MIL-125-CS beads reached equilibrium in 180 min, achieving a maximum Pb^2+^ adsorption of 407.50 mg g^−1^ at ambient temperatures. Furthermore, reusability tests showed a minor loss in the Pb^2+^ removal capacity of MIL-125-CS after five cycles of reuse, demonstrating that this is a promising application for the adsorptive removal and recovery of heavy metal pollutants.

**Figure 5:**
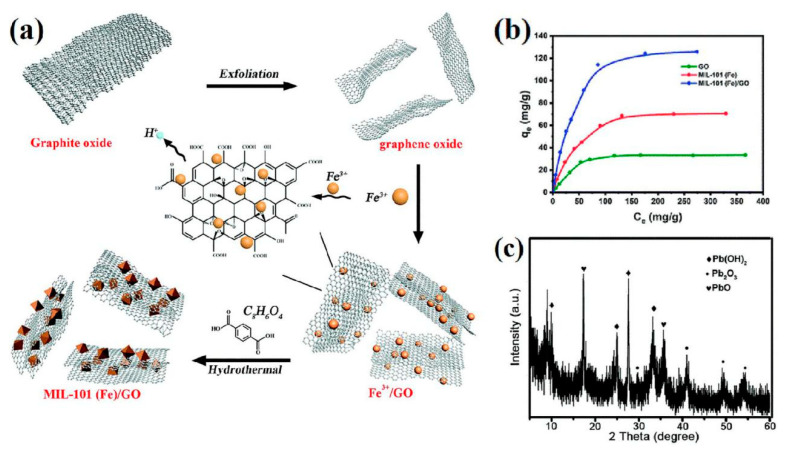
(**a**) Schematic illustration of the synthesis of MIL-101(Fe)/GO with a sandwich structure via a hydrothermal method; (**b**) Pb^2+^ adsorption isotherms for GO, MIL-101(Fe) and MIL-101(Fe)/GO at room temperature; (**c**) XRD pattern of MIL-101(Fe)/GO after Pb^2+^ adsorption. Reproduced with permission from [83]. Copyright Royal Society of Chemistry, 2019.

The instability of MOFs often limits their development and application for the removal of heavy metal ions. To address this issue, Lu et al. [83] proposed a hydrothermal method to modify Fe-based MOF MIL-101(Fe) with graphene oxide (GO), forming a typical sandwich structure (Figure 5a), improving the materials water stability and adsorption capacity. As a result of the large specific surface area and abundance of active sites, MIL-101(Fe)/GO exhibited a high adsorption capacity of 128.6 mg g^−1^ (Figure 5b) and an efficient adsorption rate within 15 min, for the removal of Pb^2+^ from aqueous solution. As shown in Figure 5c, the XRD patterns of MIL-101(Fe)/GO before and after Pb^2+^ adsorption revealed a basic adsorption mechanism of ion exchange, in which Pb^2+^ is transformed into oxides and hydroxides via bonding with the oxygen or hydroxy groups of MIL-101(Fe)/GO, respectively.

**Figure 6:**
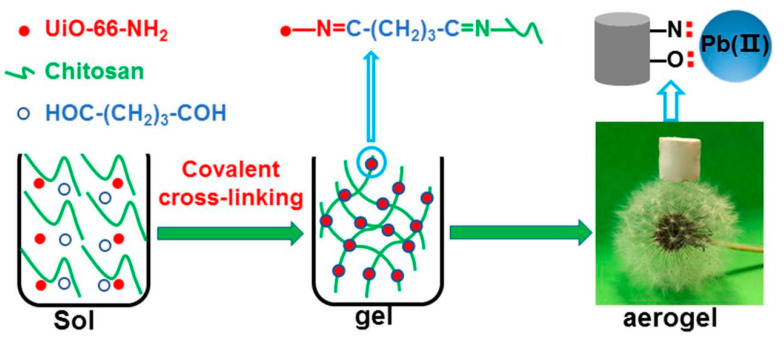
Synthesis of UiO-66–NH_2_–CS aerogel monolith (UNCAM). Reproduced with permission from [85]. Copyright Academic Press Inc., 2020.

A novel metal organic framework, UiO-66–resorcyl aldehyde (RSA), was prepared via the post-modification of UiO-66–NH_2_ (UiO-66 with amino) with resorcyl aldehyde, for the efficient and selective separation and removal of Pb^2+^ from aqueous media [84]. Results showed that Pb^2+^ could be selectively adsorbed by UiO-66–RSA, with a maximum Pb^2+^ adsorption amount of 189.8 mg g^−1^ at an optimal pH of 4. The adsorption process was monolayer chemisorption and the results of the XPS analysis suggested that the adsorption mechanism was a complexation reaction between hydroxyl/nitrogen-containing groups and Pb^2+^. The results of this study indicated that UiO-66–RSA has great potential for application in the removal of Pb^2+^ from aqueous solutions. Recently, a novel method to prepare a hierarchically porous UiO-66–NH_2_–CS aerogel monolith (UNCAM) was reported, with synthesis via covalent crosslinking between UiO-66–NH_2_ particles and CS as the supporting material (Figure 6) [85]. The highest Pb^2+^ adsorption capacity of UNCAM reached 102.03 mg g^−1^, with the Pb^2+^ adsorption exhibiting good fitting with both the pseudo-second-order model and Langmuir model. The adsorption mechanism of coordination interactions between N and Pb^2+^ was confirmed, with O also playing a synergistic role in adsorption to a certain degree. In general, this production pathway can efficiently convert MOF powders into a shapeable form and is highly promising for application in wastewater treatment systems.

Table 4 summarizes the reported data on the Pb^2+^ adsorption by several MOF-based materials, displaying the relatively fast adsorption kinetics overall. The introduction of different functional groups (such as amino groups, thiol groups and oxygen-containing groups) into the structure of MOFs has been shown to be highly beneficial for effective Pb^2+^ removal. However, further studies should explore the interaction mechanisms in more detail, using experimental and theoretical approaches.

### 2.5. Adsorptive Removal of Cadmium

**Figure 7:**
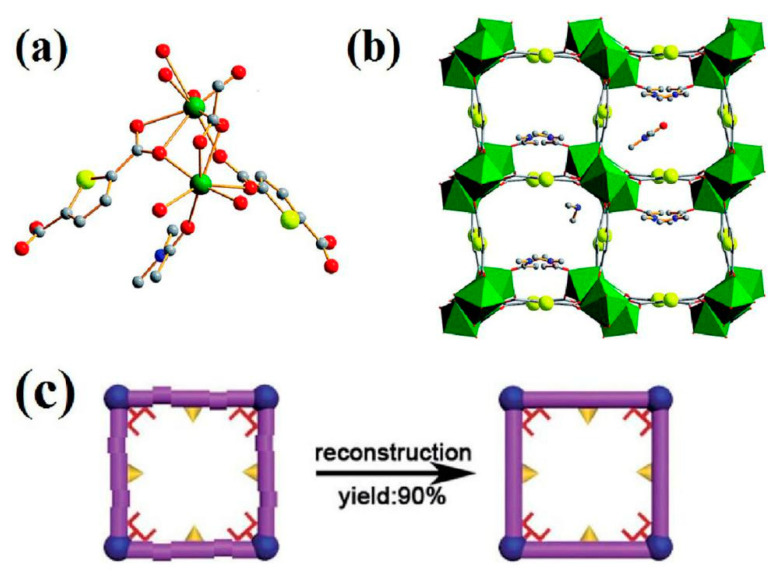
(**a**) The coordination environment of FJI-H9; C, grey; N, blue; O, red; S, yellow; Ca, green; H was omitted for clarity; (**b**) The 3D framework of FJI-H9. Sulfur atoms are highlighted as yellow balls, and Ca atoms are highlighted as green polyhedral; (**c**) In situ regeneration of the used framework. Reproduced with permission from [87]. Copyright Royal Society of Chemistry, 2016.

There have been few reports on the adsorption of cadmium ions by MOFs to date. The functionalization of MOF with sulfonic acid, generated Cu_3_(BTC)_2_–SO_3_H, exhibited a relatively low cadmium uptake capacity of 88.7 mg g^−1^ with fast adsorption kinetics [86]. Notably, the removal efficiency was not influenced by the presence of interfering metal ions (Na^+^, Mg^2+^, Ca^2+^, Pb^2+^, Cu^2+^, and Ni^2+^) mainly due to the –SO_3_H functionalization. This functional material can be readily regenerated and recycled after six cycles of reuse without significant loss of adsorption capacity. The main mechanism for Cd^2+^ adsorption was the chelation between Cd^2+^ and –SO_3_H groups. A highly effective MOF FJI-H9 has been developed for the reversible uptake of Cd^2+^, with the framework structure of FJI-H9, shown in Figure 7a,b [87]. The adsorption capacity of FJI-H9 reached 225 mg g^−1^, which surpassed other cadmium ion adsorbents due to an unusual synergy between active sites and the confined cavity. FJI-H9 was found to be effective in the presence of a diverse range of coexisting metal ions (Cd^2+^, Hg^2+^, Ca^2+^, Mg^2+^, Co^2+^, Ni^2+^, Mn^2+^, Zn^2+^, Fe^2+^ and Pb^2+^). FJI-H9 can be reused after multiple cycles of usage, although its efficiency is reduced. However, based on the reversibility of the coordination bond, the used FJI-H9 framework can be regenerated in situ (Figure 7c). In addition, FJI-H9 was able to detect Cd^2+^ effectively and quickly by fluorescence quenching, due to the coordinated dimethylacetamide (DMA) molecules and free DMA molecules stacked in the 1D channels of the FJI-H9 framework, allowing the easy intermolecular energy transfer between the FJI-H9 framework and Cd^2+^.

Amino groups (−NH_2_) have lone pair electrons and act as a Lewis base, forming coordinate bonds with the heavy metal ions (Lewis acids). Based on this, −NH_2_ functionalized Zr-MOFs were prepared via a rapid microwave-promoted synthesis method, for use in the adsorptive removal of Cd^2+^ [88]. A high Cd^2+^ adsorption capacity of 177.35 mg g^−1^ can be achieved at an initial Cd^2+^ concentration of 40 mg L^−1^ under the optimum conditions of pH 6 and 30 °C, after 2 h of adsorption. The adsorption mechanism was confirmed as a coordination interaction between the amino group (−NH_2_) and Cd^2+^. Another metal–organic framework, TMU-16–NH_2_, exhibited a maximum Cd^2+^ adsorption capacity of 126.6 mg g^−1^ [89]. The kinetics of Cd^2+^ adsorption by TMU-16–NH_2_ fitted well to the pseudo-second-order model and 98.91% of Cd^2+^ was adsorbed in only 30 min of contact time. Data indicated that the amino-groups of TMU-16–NH_2_ are favorable Cd^2+^ binding sites and that coordinate bonds formed between Cd^2+^ and −NH_2_ groups.

Recently, an eco-friendly *γ*-cyclodextrin MOF-based nanoporous carbon (*γ*-CD MOF-NPC) material was synthesized, exhibiting excellent adsorption performance and achieving over 90% of Cd^2+^ removal within one minute [90]. The maximum adsorption capacity was calculated to be 140.85 mg g^−1^. According to the FTIR spectra and zeta potential analysis results, the adsorption was found to be mainly due to the ion exchange with oxygen-containing groups on the surface of *γ*-CD MOF-NPC via electrostatic interactions. Furthermore, cytotoxicity experiments indicated that *γ*-CD MOF-NPC exerted almost no toxicity when applied at a concentration lower than 50 mg L^−1^, providing valuable guidance for its practical application in water treatment. A MOF composite nanofiber adsorbent, polyacrylonitrile (PAN)/chitosan/UiO-66–NH_2_, was found to have a high Cd^2+^ adsorption capacity of 415.6 mg g^−1^. UiO-66–NH_2_ was synthesized via a microwave heating method and incorporated into electrospun PAN/chitosan nanofibrous membranes for the removal of Cd^2+^ in adsorption and membrane filtration processes [91]. During the membrane filtration process, the PAN/chitosan/UiO-66–NH_2_ nanofiber was attached to PVDF nanofibers to produce the PVDF/PAN/chitosan/UiO-66–NH_2_ nanofibrous membranes. The high water flux and high metal ion removal capability of the PVDF/PAN/chitosan/UiO-66–NH_2_ membrane within 18 h of filtration time, showed that this material has high potential for the removal of metal ions from aqueous solutions. The adsorption characteristics of Cd^2+^ on the reported MOF-based materials are outlined in Table 5.

## 3. Conclusions and Prospects

As a new porous crystalline material, MOFs exhibit excellent adsorption performance, effectively removing heavy metal ions from aqueous solution. Compared with other adsorption materials, MOFs have many advantages such as large surface area, high porosity, adjustable apertures, diverse structures, open metal sites and chemical modifiability.

The recent research advances on the removal of heavy metal ions by MOFs and MOF-based materials were provided. As recently developed adsorbents for the removal of toxic metal ions, MOFs and MOF-based materials exhibit the advantages of fast adsorption kinetics, high adsorption capacities, excellent selectivity and reusability. The proposed mechanisms of adsorption for MOFs and MOF-based composites were generally based on electrostatic interactions, ion exchange and a combined adsorption–reduction process, with strong coordination between the target ions and the functional binding groups (e.g., hydroxyl, thiol, amide substituents and other oxygen-containing groups). Post-synthesis modification, in situ synthesis with linkers containing substituents, and hybridization with specific functional materials, have all been proven to be efficient strategies for the improvement of the adsorption performance and selectivity of MOFs and MOF-based composites.

Although great progress has been achieved in recent years, there remains a number of challenges and issues that need to be addressed: (1) some MOFs have a narrow active pH range and exhibit poor water stability, which limits their practical application; (2) the solution conditions (e.g., pH, ionic strength, absorbent dose, and the coexistence of competitive ions) have been found to strongly affect the surface properties of MOFs and the adsorption behavior of toxic metal ions, although detailed information has not been provided adequately in many known reports; (3) the synthesis of many MOFs relies on the use of expensive ligands; (4) most MOFs are synthesized in organic solvents by solvothermal methods, such as *N*,*N*-dimethylformamide (DMF) and methanol, which leads to secondary pollution; (5) the pore size of most reported MOFs is limited to the micropore range, which may hinder the mobility of large metal ions within the framework; and (6) many MOFs cannot currently be produced quickly, in large quantities, or at a low cost, which also restricts the prospects for the practical application of MOFs.

In view of these limitations, future research should include the following aspects: (1) MOFs with good pH adaptability and stability should be further explored for practical application; (2) the adsorption behaviors and mechanisms of MOFs need to be studied in more detail; (3) low-cost organic ligand alternatives would be beneficial; (4) the environmental impact of the used solvents should be considered and the appropriate methods selected to allow the recycling of organic solvents to mitigate secondary pollution. In addition, alternative synthesis methods with a low environmental impact should be developed; (5) MOF structures should be modified to fully utilize the abundant adsorption/conversion sites distributing throughout the fine structure; (6) developing more cost-effective design strategies to promote the industrial application of MOFs.

Despite the problems and challenges that remain in this area, MOF materials show great potential in the adsorption and removal of heavy metal ions. Moreover, the real contaminated aqueous systems urgently need us to address the important issues concerning the techniques of MOF application as adsorbents. Overall, it can be expected that the practical application of MOF materials for water purification will soon become possible with further research advancements.

## Figures and Tables

**Table 1:** Arsenic adsorption data for the reported Metal–organic frameworks (MOFs) and MOF-based materials.

Adsorbates	MOFs/MOF-Based Materials	Adsorption Capacity (mg g^−1^)	Equilibrium Time	pH	Selectivity	Reusability	Mechanism	Ref.
As(V)	ZIF-8	76.5	7 h	4	vs. Cl^−^, F^−^, NO_3_^−^, SO_4_^2^^−^, PO_4_^3^^−^	Reusable	Inner-sphere complex	[44]
	ZIF-8	90.92	10 h	—	—	Reusable	Ion exchange	[45]
	MIL-53(Fe)	21.27	90 min	5	—	—	Lewis acid–base and electrostatic interactions	[49]
	Fe-BTC	12.3	10 min	4	—	—	Ion exchange	[50]
	MIL-53(Al)	105.6	11 h	8	vs. CO_3_^2^^−^, Cl^−^, NO_3_^−^, SO_4_^2^^−^	—	Electrostatic attraction, hydrogen bond	[51]
	MIL-100(Fe)	110	1 h	—	vs. Cl^−^, SO_4_^2^^−^, NO_3_^−^, CO_3_^2^^−^	Reusable	Coordination	[52]
	MOF-808	24.83	30 min	4	—	Reusable	Ion exchange	[55]
	UiO-66	303.34	48 h	2	vs. CO_3_^2^^−^, Cl^−^, NO_3_^−^, SO_4_^2^^−^	—	Ion exchange	[56]
As(III)	Cubic ZIF-8	122.6	10 h	8.5	—	—	Ion exchange	[46]
	Leaf-shaped ZIF-8	108.1	10 h	8.5	—	—	Ion exchange	[46]
	Dodecahedral ZIF-8	117.5	10 h	8.5	vs. CO_3_^2^^−^, PO_4_^2−^, SO_4_^2^^−^, Cl^−^	Reusable	Ion exchange	[46]
	*β*-MnO_2_@ZIF-8	140.27	24 h	7	—	—	Oxidation	[47]
As(V)/As(III)	ZIF-8	60.03/49.49	7 h/13 h	7	vs. SO_4_^2^^−^, PO_4_^2−^, CO_3_^2^^−^, NO_3_^−^	—	Ion exchange	[48]
	Fe_3_O_4_@MIL-101(Cr)	80.0/121.5	24 h/24 h	7	vs. various oxyanions, Ca^2+^, Mg^2+^	—	Ion exchange	[53]
	CoFe_2_O_4_@MIL-100(Fe)	114.8/143.6	2 min/2 min	4–10	vs. various oxyanions, Congo red, humic acid	—	Ion exchange/hydrogen bonding	[54]

Note: iron-1,3,5-benzenetricarboxylic polymers (Fe-BTC).

**Table 2:** Chromium adsorption data for the reported MOFs and MOF-based materials.

Adsorbates	MOFs/MOF-Based Materials	Adsorption Capacity (mg g^−1^)	Equilibrium Time	pH	Selectivity	Reusability	Mechanism	Ref.
CrO_4_^2–^	{[Ag_8_(tz)_6_](NO_3_)_2_·6H_2_O}_n_	37	NA	6	vs. NO_3_^−^, CO_3_^2^^−^	—	Ion exchange	[57]
Cr_2_O_7_^2−^	FIR-53	74	10 min	—	vs. Cl^−^, Br^−^, NO_3_^−^	Reusable	Ion exchange	[58]
	FIR-54	103	30 min	—	—	Reusable	Ion exchange	[58]
	Fe-gallic acid MOF	1709.2	72 h	4	vs. various ions	Limited	Multiple	[59]
	MP@ZIF-8	136.56	16 h	5	—	—	Adsorption reduction	[60]
	MOR-1-HA	242–280	3 min	3	vs. Cl^−^, Br^−^, CO_3_^2^^−^, SO_4_^2^^−^	Reusable	Ion exchange	[61,62]
	MOR-1 (protonated)	247–321	NA	3	—	—	Ion exchange	[61,62]
	MOR-1 (non-protonated)	267	>1 h	3	—	—	Ion exchange	[61,62]
	MOR-2-HA	162.8	1 min	3	—	Reusable	Ion exchange	[63]

Note: Fujian Institute of Research (FIR); magnetic polydopamine (MP); metal organic resin (MOR); alginic acid (HA).

**Table 3:** Mercury adsorption data for the reported MOFs and MOF-based materials.

MOFs/MOF-Based Materials	Adsorption Capacity (mg g^−1^)	Equilibrium Time	pH	Selectivity	Reusability	Mechanism	Ref.
{Ca^II^Cu^II^_6_[(*S*,*S*)-methox]_3_ (OH)_2_(H_2_O)}·16H_2_O	900	<200 min	7	vs. CH_3_Hg^+^	—	Binding with S	[64]
{Cu_4_^II^[(*S*,*S*)-methox]_2_}·5H_2_O	1 HgCl_2_ per formula	15 min	7	vs. various cations	Reusable	Binding with thiol	[65]
FJI-H12	439.8	<50 min	7	vs. Mn^2+^, Ba^2+^, Ni^2+^, Cd^2+^	Reusable	Binding with SCN^−^	[66]
Zr-DMBD	171.5	10 min	6	vs. various cations	Reusable	Coordination with S^2−^	[67]
UiO-66–SH	785	20 min	4	vs. various cations	Reusable	Interact with thiol	[68]
[Zn(hip)(L)]·(DMF)(H_2_O)	250	1 h	5	—	—	Contact with C=O	[69]
MIL-101-thymine	59.28	300 min	6	vs. various cations	Limited	Coordination with N	[70]
NH_2_–ML–53(Al)	153.85	60 min	6	—	Reusable	Coordination with –NH_2_	[71]
TMU-32S–65%	1428	17 min	4.4	vs. various cations	Reusable	Electrostatic interaction, chelation	[72]

Note: bis[(S)-methionine]oxalyl diamide (methox); *N*,*N*-dimethylformamide (DMF); Tarbiat Modares University (TMU).

**Table 4:** Lead adsorption data for the reported MOFs and MOF-based materials.

MOFs/MOF-Based Materials	Adsorption Capacity (mg g^−1^)	Equilibrium Time	pH	Selectivity	Reusability	Mechanism	Ref.
ED-MIL-101(Cr)	81.09	30 min	6	vs. various cations	Limited	Specific binding	[76]
MIL-53(Al) MFC	492.4	120 min	—	—	—	Ion exchange	[77]
Melamine-MOFs	205	120 min	6	—	—	Coordination interaction	[78]
Thiol-functionalized Fe_3_O_4_@Cu_3_(btc)_2_	215.05	120 min	5.92	vs. various cations	Reusable	Binding with thiol	[79]
MCNC@Zn-BTC	558.66	30 min	5.45	vs. Cu^2+^, Zn^2+^, Cd^2+^	Reusable	—	[80]
{[Cd(ADB)L_2_]·1.5 DMF·2H_2_O}_n_	63.052	5 min	7	vs. various cations	Reusable	Pb/O=C−O interaction	[81]
MIL-125-CS	407.50	180 min	6	—	Reusable	—	[82]
MIL-101(Fe)/GO	128.6	15 min	6	—	Reusable	Ion exchange	[83]
UiO-66–RSA	189.8	150 min	4	vs. various cations	Reusable	Complexation reaction	[84]
UNCAM	102.03	300 min	5	—	Reusable	Coordination interaction	[85]

Note: ethylenediamine (ED); magnetic framework composites (MFCs); magnetic cellulose nanocrystals (MCNC); Zn–benzene-1,3,5-tricarboxylic acid (Zn–BTC); chitosan (CS); UiO-66–NH_2_–CS aerogel monolith (UNCAM).

**Table 5:** Cadmium adsorption data for the reported MOFs and MOF-based materials.

MOFs/MOF-Based Materials	Adsorption Capacity (mg g^−1^)	Equilibrium Time	pH	Selectivity	Reusability	Mechanism	Ref.
Cu_3_(BTC)_2_–SO_3_H	88	10 min	6	vs. various cations	Reusable	Ion exchange	[86]
FJI-H9	225	NA	—	vs. various cations	Reusable	Cd–O interactions	[87]
NH_2_-functionalized Zr-MOFs	177.35	120 min	6	—	—	Coordination interaction	[88]
TMU-16–NH_2_	126.6	90 min	6	—	—	Ion exchange	[89]
*γ*-CD MOF-NPC	140.85	60 min	7	vs. Na^+^, Ca^2+^, Mg^2+^, NO_3_^−^, SO_4_^2^^−^, Cl^−^	—	Ion exchange	[90]
PAN/chitosan/UiO-66–NH_2_	415.6	60 min	6	—	Reusable	—	[91]

Note: *γ*-cyclodextrin (*γ*-CD); nanoporous carbon (NPC); polyacrylonitrile (PAN).
